# Co-testing a single sputum specimen for TB and SARS-CoV-2

**DOI:** 10.5588/ijtld.22.0404

**Published:** 2023-02-01

**Authors:** L. P. Genade, T. Kahamba, L. Scott, S. Tempia, S. Walaza, A. David, W. Stevens, K. Hlongwane, A. von Gottberg, M. Du Plessis, J. Kleynhans, C. Cohen, N. A. Martinson

**Affiliations:** 1 Perinatal HIV Research Unit (PHRU), School of Pathology, Faculty of Health Sciences, University of the Witwatersrand, Johannesburg, South Africa; 2 Department of Molecular Medicine and Haematology, School of Pathology, Faculty of Health Sciences, University of the Witwatersrand, Johannesburg, South Africa; 3 Centre for Respiratory Diseases and Meningitis, National Institute for Communicable Diseases, Division of National Health Laboratory Service, Sandringham, South Africa; 4 School of Public Health, Faculty of Health Sciences, University of the Witwatersrand, Johannesburg, South Africa; 5 National Priority Programme, National Health Laboratory Services, Johannesburg, South Africa; 6 School of Pathology, Faculty of Health Sciences, University of the Witwatersrand, Johannesburg, South Africa; 7 Johns Hopkins University Center for TB Research, Baltimore, MD, USA

Dear Editor,

The COVID-19 pandemic has caused devastating disruption to diagnostic services for TB.^1^ During the COVID-19 lockdowns in South Africa, the number of Xpert^®^ MTB/RIF Ultra (Ultra) (Cepheid, Sunnyvale, CA, USA) tests were reduced by 48%, leading to a decline by 33% in individuals diagnosed with TB.^2^ Because TB and COVID-19 have symptoms in common, we posited that collecting a single specimen for simultaneous testing might allow us to identify patients with TB at a time when testing is focused on SARS-CoV-2.^3^

We nested a study of paired testing for TB and SARS-CoV-2 from a single sputum specimen as part of a larger study of household transmission for SARSCoV-2.^4^ Participants were enrolled at primary health clinics in Soweto. To be eligible for the parent study, individuals had to have at least two people in their household; be the first in the household with symptoms of COVID-19 for ≤7 days; and have documented HIV status. Nasopharyngeal swab and sputum specimens were collected from consenting participants from 28 July to 30 September 2021. Those unable to spontaneously expectorate a sputum were asked to breathe deeply several times, cough repeatedly and then spit into the specimen container; this had to be repeated until ≥3 ml of sputum was collected. Nasopharyngeal swabs were tested for SARS-CoV-2 at the National Institute for Communicable Diseases, Johannesburg, South Africa. Each sputum specimen was subjected to both Ultra and Xpert Xpress SARS-CoV-2 (Xpress SARS-CoV-2) testing at a TB research laboratory. The TB laboratory was blinded to the nasopharyngeal SARS-CoV-2 test result. The study was approved by the local Ethics Committee.

We pre-screened 472 individuals; of the 59 who consented we obtained a nasopharyngeal and a sputum specimen from 57 (the latter using a swab capture method).^5^ The swab swirled in sputum was tested using Xpress SARS-CoV-2 and the residual sputum was used for Ultra testing. Three participants (3/57, 5.3%) tested positive for *Mycobacterium tuberculosis* (MTB) on the Ultra assay; one had a trace result (the lowest semi-quantitative result) and was subsequently confirmed using liquid culture as MTB. One participant was HIV co-infected, none had diabetes (Table). Of 57 sputum specimens, 11 (19.3%) were positive for SARS-CoV-2 on either nasopharyngeal and/or sputum SARS-CoV-2 testing; 8/11 were positive on both assays. Sputum Xpert Xpress detected an additional two cases of SARSCoV-2, while nasopharyngeal testing found one additional case. No participant tested positive for both SARS-CoV-2 and MTB.

**Table:** Characteristics of participants co-tested for SARS-CoV-2 and Mycobacterium tuberculosis

Variable	Overall (*n* = 57) *n*/*N* (%)	*Mycobacterium tuberculosis* (*n* = 3) *n*/*N* (%)	SARS-CoV-2 (*n* = 11) *n*/*N* (%)
Age, years, median [IQR]	38.0 [29.0–48.0]	46.0 [45.0–56.0]	47.0 [39.0–65.0]
Women	48/57 (84.2)	1/3 (33.3)	10/11 (90.9)
HIV-infected	7/57 (12.3)	1/3 (33.3)	4/11 (36.4)
Diabetes	3/54 (5.6)	0/3 (0.0)	1/9 (11.1)
BMI, kg/m^2^, median [IQR]	27.0 [23.1–34.2]	19.4 [16.9–19.9]	30.7 [23.7–34.8]
Respiratory symptoms	57/57 (100.0)	3/3 (100.0)	11/11 (100.0)
Duration, days, median [IQR]	3.00 [2.00–3.0]	3.00 [2.00–4.0]	3.00 [2.00–3.0]
Cough	51/57 (89.5)	3/3 (100.0)	10/11 (90.9)
Fever or chills	25/57 (43.9)	1/3 (33.3)	3/11 (27.3)
Night sweats	17/57 (29.9)	2/3 (66.7)	1/11 (9.9)
Weight loss	5/57 (8.8)	1/3 (33.33)	2/11 (18.2)

IQR = interquartile range; BMI = body mass index.

Despite restrictive eligibility criteria for the parent study and our small sample size, our findings raise concerns about missed TB diagnoses during the COVID-19 pandemic. Our data suggest that consideration should be given to co-testing for TB in high TB burden settings. Although we make no recommendations or conclusions on the use of a sputum sample for co-testing, especially as collection is aerosol generating, the three individuals with laboratory-confirmed MTB in this study would likely not have been tested for TB. No costing data were collected. Other single sample types, which are easier and safer to collect, should be evaluated help diagnose missing TB patients. Earlier diagnosis of both TB and COVID-19 may reduce morbidity, mortality and transmission.
